# Signatures of Gait Movement Variability in CKD Patients Scheduled for Hemodialysis Indicate Pathological Performance Before and After Hemodialysis: A Prospective, Observational Study

**DOI:** 10.3389/fmed.2021.702029

**Published:** 2021-07-28

**Authors:** Damiano D. Zemp, Olivier Giannini, Pierluigi Quadri, Marco Rabuffetti, Mauro Tettamanti, Eling D. de Bruin

**Affiliations:** ^1^Department of Health Sciences and Technology, Institute of Human Movement Sciences and Sport, ETH Zurich, Zurich, Switzerland; ^2^Geriatric Service, Ente Ospedaliero Cantonale, Ospedale Regionale della Beata Vergine, Mendrisio, Switzerland; ^3^Department of Internal Medicine, Ente Ospedaliero Cantonale, Mendrisio, Switzerland; ^4^Service of Nephrology, Ente Ospedaliero Cantonale, Ospedale Regionale della Beata Vergine, Mendrisio, Switzerland; ^5^Faculty of Biomedical Sciences, Università della Svizzera Italiana, Lugano, Switzerland; ^6^IRCCS Fondazione Don Carlo Gnocchi, Milan, Italy; ^7^Department of Neuroscience, Istituto di Ricerche Farmacologiche Mario Negri IRCSS, Milan, Italy; ^8^Department of Neurobiology, Care Sciences and Society, Karolinska Institute, Stockholm, Sweden; ^9^OST – Eastern Swiss University of Applied Sciences, Department of Health, St. Gallen, Switzerland

**Keywords:** chronic kidney disease, end stage renal disease, hemodialysis, physical activity, gait, variability, dual-task costs

## Abstract

**Background:** The frailty status of hemodialysis patients is well-known, but the role of the therapy in the frailty process is not yet clear. Nowadays gait analysis in nephrology is neglected, although gait performance is known to be related to frailty and kidney function. We hypothesized that gait quality and physical activity level is already affected before, and does not change because of the start of hemodialysis.

**Methods:** Fourteen patients (72.3 ± 5.7 years old) in a pre-dialysis program underwent an instrumental gait analysis and their physical activity was monitored for a week. This protocol was repeated 3, 6, 12, and 24 months after the first hemodialysis session.

**Results:** At baseline, our sample showed a conservative gait with pathologic gait variability, high dual-task cost, and a sedentary lifestyle. No statistically significant change was found in any parameter in the analyzed period, but there was a tendency toward an improvement of gait quality and physical activity in the first year of treatment, and a decline in the second year.

**Conclusion:** Elderly patients in the pre-dialysis stage show a conservative gait, however variability was in a pathological range and did not change post-hemodialysis. This hints toward changes in the central nervous system due to the kidney disease. This finding suggests the importance of gait analysis in the early stages of renal disease in the diagnosis of changes in the nervous system due to kidney failure that affect gait. Early detection of these changes would potentially allow a prevention program tailored to this population to be developed.

## Introduction

Hemodialysis (HD) is the most frequent renal replacement therapy (RRT) for patients with end stage renal disease (ESRD). About 2 million patients are receiving HD worldwide (1), a quarter of them in Europe (2). Switzerland, with a population of about 8 million inhabitants, registered in 2015 about 4,500 patients, with a mean age of 68 years on HD (3). With the aging of the population and the improvement of RRT quality, the number of elderly people on HD is going to increase in the near future. The higher frailty status of ESRD patients compared to the general population is widely described (4), and can partially be explained by the degenerative nature of the chronic kidney disease (CKD) that leads to an increased frailty status, with the worsening of kidney function through the years (5). Apart from this, cerebrovascular disorders induced by the HD (6, 7) and a reduction of daily physical activity (PA)—caused by the time-consuming therapy (8, 9)—can additionally negatively influence the frailty process despite the vital importance of the treatment. However, how the frailty process evolves after renal function stabilization through HD, and the role of HD in this process, is not yet fully understood (10–12).

Slow gait speed, low physical activity, unintentional weight loss, exhaustion, and muscle weakness, the five Cardiovascular Health Study (CHS) frailty index criteria, are the most used criteria to classify individuals as prefrail or frail (13). Of these criteria gait speed is one of the strongest predictors for adverse outcomes such as falls, impaired mobility or hospitalization (14–16).

While gait speed is an important indicator for the general health status, and can predict a clinical decline (17–21), other characteristics of gait are more suitable for the analysis of neurocognitive factors (22). The coefficient of variation (CV) of the stride is reported to be associated with executive function (22–24), and can discriminate between healthy and pathological gait (25, 26). Gait regularity extrapolated from the trunk movement can describe the walking pattern of the elderly or people with orthopedic or neurological disturbances (27–30). Moreover, dual-task cost (DTC) of gait can indicate a higher fall risk (31–38).

Instrumental gait analysis that assesses other aspects apart from speed is becoming increasingly part of the clinical practice in geriatrics and neurology, where screening for fall risk and frailty is an important factor (39, 40). However, this is so far rather neglected in the CKD population (41), despite this population showing a comparable functional decline (42–45). In fact, the only author known to us who analyzed gait instrumentally under single- and dual-task conditions in HD patients, found significant differences, not only in gait speed, but also in other spatio-temporal parameters, in DTC and in variability (46, 47). These studies confirm the existence of gait changes related to the frailty of HD patients; however, it remains unexplained whether there is a relation between HD and changes in gait.

This study, therefore, aimed to prospectively describe the evolution of spatio-temporal parameters, variability, DTC of gait, and daily PA in patients in a pre-dialysis program scheduled for HD. ESRD patients were followed from a few months before the start of RRT to 2 years after starting HD. We assumed that a better understanding of the processes that lead to frailty in HD patients could be useful for developing specific preventive strategies for this population.

## Methods

### Study Design

This prospective longitudinal observational study is a multicentric project in an ambulant setting of CKD patients with ESRD. In this publication we focus on the instrumental gait analysis that includes a gait analysis in a laboratory context, and a real-life monitoring of PA. When the medical doctor started to plan the beginning of HD within the next 6 months, the patient was called for the baseline assessment. During the first 2 years of the RRT, another four visits where organized: at 3, 6, 12, and 24 months. The assessments took place at the dialysis center where the participant received treatment at least 24 h after the end of an HD session.

### Participants

The patients were recruited between 2015 and 2018 in three HD units of the nephrology department of the multicentric public hospital of Canton Ticino—Switzerland (Ente Ospedaliero Cantonale in the towns of Mendrisio, Lugano and Bellinzona) and from the private dialysis center Nefrocure in Lugano.

The baseline assessment of the first patient took place in January 2015, whereas the final follow-up assessment of the last patient took place in June 2020. Inclusion criteria were (a) CKD 5 (eGFT^1^ <20 ml/min with eligibility criteria for a HD program), (b) ability to understand information for executing assessments, (c) ability to walk autonomously. Exclusion criteria were: (a) instable or preterminal health status (e.g., recent surgery, ongoing oncological treatment) (b) diagnosis of dementia [Clinical Dementia Rating Scale ≥ 1 (48)], (c) diagnosis of depressive syndromes.

Patients with ESRD were asked by their nephrologist about their interest in participating in the longitudinal study. In the case they accepted, their name and phone number were sent to the principal investigator who contacted them for the baseline visit. During this first visit, before starting with the assessments, inclusion and exclusion criteria were checked, the patient was informed about the study in an oral and written form and requested to sign the written informed consent. Patients who didn't start HD as expected were re-analyzed in 6 months intervals till the start of the RRT.

### Instruments and Protocols

At baseline, general characteristics were recorded: age, gender and BMI.

In the laboratory, the gait analysis was performed on a 14 m pathway via a triaxial accelerometer (DynaPort MiniMod, McRoberts, The Hague, NL) affixed to the lower trunk—between the left and the right spina iliaca posterior superior—by an elastic belt. This device is designed for clinical gait analysis in a laboratory setting (49). Spatio-temporal gait parameters were calculated using the inverted pendulum model (50, 51), and result in valid and reliable measurements in patients with chronic conditions (52, 53).

The intra-subject analysis of the gait variability was defined on the one hand using the CV (the ratio of standard deviation and mean) for stride time and stride length, and on the other hand by regularity of locomotion calculation, based on the autocorrelation analysis of the acceleration module (norm of the acceleration vector) (54). To register gait speed at steady state walking—a fundamental requisite for calculating valid variability and regularity—the first and the last 2 meters were excluded from the data analysis. Each participant walked two times at self-selected speed over the pathway (single-task), and two times while counting down from 100 in steps of three (dual-task). Depending on the step length of the participants, between 15 and 35 strides were used for calculating gait parameters. The participants were not allowed to use a walking aid.

For gait monitoring, participants wore a pedometer (Step Watch^TM^, Modus, Washington DC, USA) for nine consecutive days (in order to get 7 days monitored for 24 h) on the right ankle (55), which measured the number of right-leg steps and recorded in 1-min intervals. In order to have an output to be compared with international normative data, the number of steps was doubled (56).

### Variables

The following spatio-temporal parameters were extracted from the gait analysis: gait speed, cadence, step, and stride time (ST), step, and stride length (SL). CV in both temporal and spatial domain were calculated for step and stride. In addition, gait regularity was calculated. Because renal disease patients exhibit major changes in the central nervous system (26, 57–60), we compared our gait variability data with optimal thresholds derived from healthy and neurological patients (26). The DTC of gait speed, cadence and SL (expected to decrease with dual-task) was determined using the formula

$$\begin{array}{l} 100\;\cdot \;\frac{\text{Single }-\text{ Task\_value }-\text{ Dual }-\text{ Task\_value}}{\text{Single }-\text{ Task\_value}} \end{array}$$

and ST (expected to increase with dual-task) from the formula

$$\begin{array}{l} 100\;\cdot \;\frac{\text{Dual }-\text{ Task\_value }-\text{ Single }-\text{ Task\_value}}{\text{Single }-\text{ Task\_value}} \end{array}$$

Since no healthy age-matched participant was enrolled, the normative data for the considered variables and indexes, are retrieved from reference literature (61).

### Statistical Methods

No power analysis was made because of the explorative character of the study and the lack of previous comparable research. The centers involved for recruitment enroll about 50 patients per year (the reference population is about 350,000 inhabitants). Based on that and excluding patients who opted for an alternative RRT as HD, and who do not follow a pre-dialysis program, we expected to recruit about 30–40 patients in the defined period (7–10 patients per year). We inserted the raw data anonymously in a database that represented the basis for the statistical analysis. For all variables listed above, descriptive statistics were calculated.

For the longitudinal data, a regression analysis by one-way repeated-measure ANOVA was made where sphericity was met. Where sphericity was not met, a non-parametric test was used. We also did a step-by-step analysis with the test for within-subject contrasts, to analyze the changes between each interval.

For comparing PA between dialysis and no-dialysis days, the paired-samples *t*-test was used.

We did not replace and did not adjust the mean and the SD in cases of missing data.

IBM SPSS Statistics 26 was used for statistical analysis and the level of significance was set at *p* ≤ 0.05.

### Ethical Aspects

We carried out the study procedures in accordance with the 1964 Declaration of Helsinki (62), and subsequent amendments. All data were collected anonymously in accordance with Swiss law (63).

The manuscript was created following the STROBE guidelines (64, 65). The checklist is available in Supplementary Material 1.

We respected all protection policies defined by the Federal and Cantonal Health Department during the visits.

## Results

### Participants

**Figure 1:**
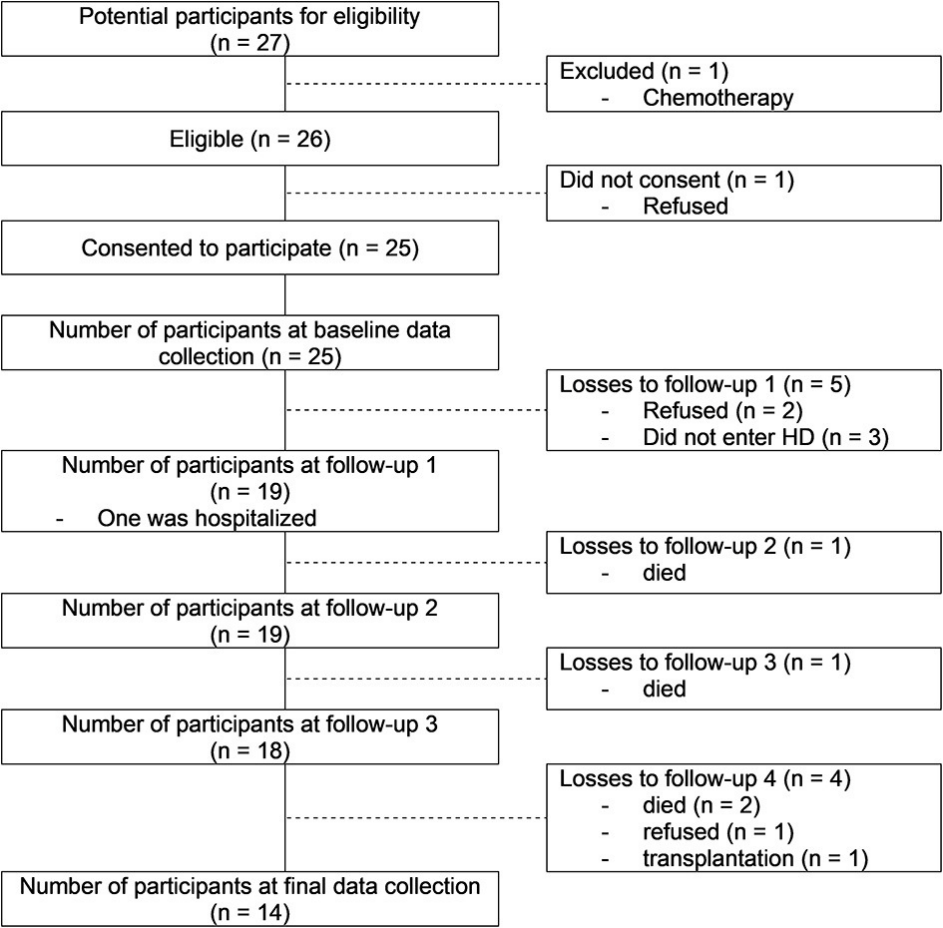
Study flow diagram.

From the 27 patients recruited from the nephrologists, 25 accepted to participate and 14 completed the scheduled assessments. Eleven participants dropped out because of retreatment (*n* = 3), death (*n* = 4), transplantation (*n* = 1) and the stabilization of the renal function (*n* = 3). The recruitment process is described in the flow diagram (Figure 1). In this study we focus on the 14 participants who completed all scheduled gait assessments. In Table 1 we summarize their general characteristics.

**Table 1:** Characteristics of the participants [mean ± SD (min–max)] by groups.

	**Completers (*n* = 14)**	**Dropouts (*n* = 11)**
Gender (M/W)	7/7	5/6
Age (years)	72.4 ± 5.4 (60–81)	76.6 ± 6.5 (64–86)
BMI (kg/m^2)^	29.7 ± 3.6 (22.6–35.1)	28.9 ± 6.5 (16.7–40.2)
Education (years)	8.2 ± 3.3 (5–17)	8.2 ± 3.8 (3–17)
Comorbidities^*^	1.6 ± 0.6 (1–3)	1.8 ± 1.3 (1–4)
Walking aid outside the house (Y/N)	4/10	3/8
Gait speed (m/s)	0.87 ± 0.28 (0.35–1.2)	1.00 ± 0.37 (0.49–1.54)

* *Cumulative Illness Rating Scale*.

### Spatio-Temporal Parameters, Variability and Dual-Task Cost of Gait

**Figure 2:**
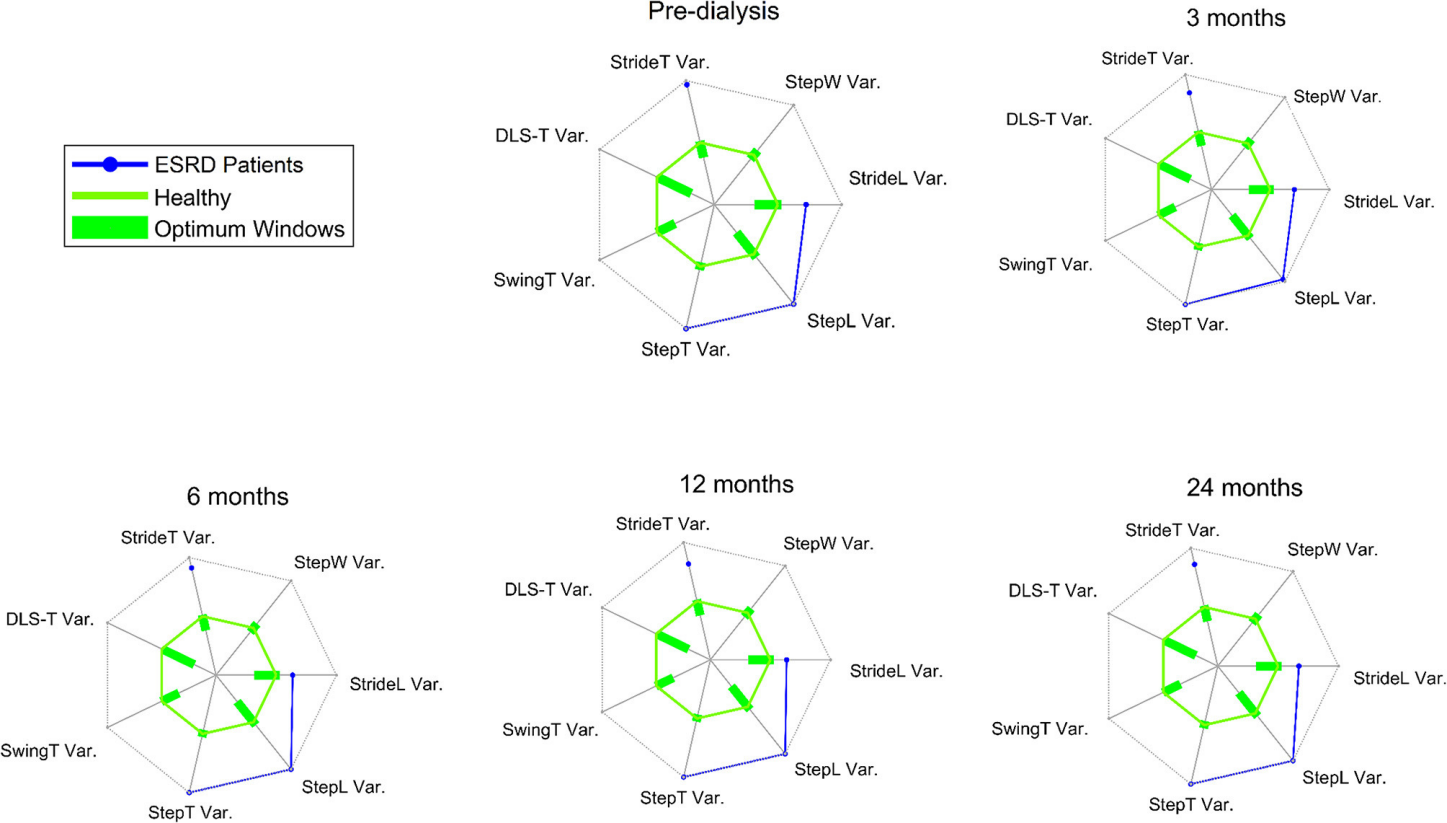
The green solid line represents the overall mean of the commonly reported gait variability parameters for healthy asymptomatic controls, obtained from studies included within the systematic review of Ravi et al. (26). The optimum windows for the gait characteristics are depicted as green bars on the different axes on the radial plot. All values are presented in standardized or z-scores. The solid axes on each of the radial plots, in gray and radiating from the center of the plot, range from −5.5 to 5.5 z-scores. In blue the measures for CKD patients. ESRD = End Stage Renal Disease; StrideT Var. = CV of stride time; StepW Var. = CV of stride width; StrideL Var. = CV of stride length; StepL Var. = CV of step length; StepT Var. = CV of step time; SwingT Var. = CV of swing time; DLS-T Var. = CV of double limb support.

In general gait speed (0.87 ± 0.28 m/s), cadence (104 ± 15 step/min), SL (1.02 ± 0.23 m) and ST (1.20 ± 0.20 s) were at the limit of normality (66–68). Variability was very high with CV values (6.66 ± 3.36 for ST and 5.53 ± 2.50 for SL) (25, 26, 61, 69) and gait regularity (0.86 ± 0.11) (30). The radar plots in Figure 2 show the comparison of the CV of several gait parameters between our sample and healthy controls extracted from a systematic review (26).

The DTC of gait speed was >20% (70, 71). Cadence, ST, and SL were less affected by the additional cognitive task and showed a DTC of about 10%.

The gait performance, variability and DTC remained stable with a tendency to improve with the beginning of HD, reaching a peak in the 12-month visit, and then decreasing in the second year (except for DTC, which remained stable). The data are reported in Table 2. The evolution of each participant is graphically represented in Supplementary Material 2.

**Table 2:** Prospective development of spatiotemporal gait parameters [mean ± SD (min–max)].

	**Pre-dialysis (*n* = 14)**	**3 months (*n* = 13)**	**6 months (*n* = 13)**	**12 months (*n* = 13)**	**24 months (*n* = 13)**	***p (F)***
**Spatio-temporal parameters**						
Gait speed (m/s)	0.87 ± 0.28 (0.35–1.20)	0.88 ± 0.28 (0.42–1.29)	0.86 ± 0.31 (0.26–1.22)	0.93 ± 0.24 (0.53–1.23)	0.87 ± 0.23 (0.36–1.11)	0.40 (0.77)
Cadence (step/min)	104 ± 15 (71–126)	106 ± 15 (73–124)	106 ± 15 (75–126)	107 ± 15 (79–127)	103 ± 14 (74–124)	0.66 (0.52)
Stride time (s)	1.20 ± 0.20 (0.96–1.71)	1.17 ± 0.19 (0.99–1.69)	1.17 ± 0.18 (0.96–1.61)	1.16 ± 0.17 (0.96–1.55)	1.20 ± 0.18 (0.98–1.65)	0.72 (0.47)
Stride length (m)	1.02 ± 0.23 (0.58–1.34)	1.12 ± 0.23 (0.80–1.58)	1.09 ± 0.25 (0.65–1.43)	1.08 ± 0.19 (0.77–1.43)	1.09 ± 0.22 (0.73–1.46)	0.14 (2.00)
Step time (s)	0.60 ± 0.10 (0.48–0.86)	0.58 ± 0.09 (0.49–0.83)	0.59 ± 0.09 (0.48–0.81)	0.58 ± 0.09 (0.48–0.77)	0.60 ± 0.09 (0.49–0.82)	0.64 (0.59)
Step length (m)	0.51 ± 0.11 (0.29–0.67)	0.56 ± 0.12 (0.40–0.79)	0.54 ± 0.13 (0.32–0.71)	0.54 ± 0.1 (0.38–0.71)	0.54 ± 0.11 (0.36–0.73)	0.15 (1.94)
**Variability**						
CV Stride time	6.66 ± 3.36 (2.16–15.45)	5.47 ± 2.76 (2.36–9.99)	6.13 ± 3.65 (2.53–14.60)	5.24 ± 2.19 (1.44–9.00)	5.66 ± 2.51 (1.57–9.79)	0.50 (0.46)
CV Stride length	5.53 ± 2.50 (2.17–10.69)	5.31 ± 1.73 (2.84–8.72)	4.41 ± 1.80 (1.66–8.79)	4.39 ± 2.64 (0.91–10.85)	4.88 ± 2.48 (1.78–11.54)	0.50 (0.86)
CV Step time	10.56 ± 4.77 (3.42–18.99)	10.01 ± 4.34 (4.07–17.47)	8.84 ± 3.72 (3.83–16.24)	9.98 ± 6.62 (1.76–22.64)	9.39 ± 3.16 (2.61–15.74)	0.36 (1.09)
CV Step length	10.63 ± 4.53 (3.82–19.89)	9.27 ± 4.27 (3.07–18.58)	10.75 ± 6.03 (4.14–26.73)	10.81 ± 7.26 (2.96–30.44)	10.61 ± 5.05 (2.72–19.9)	0.18 (1.00)
Regularity	0.86 ± 0.11 (0.64–0.99)	0.89 ± 0.07 (0.73–0.99)	0.90 ± 0.06 (0.80–0.98)	0.89 ± 0.07 (0.78–0.98)	0.87 ± 0.12 (0.52–0.95)	0.60 (0.59)
**Dual-task cost**						
Gait speed	24 ± 22 (3–94)	17 ± 8 (7–37)	19 ± 12 (4–44)	16 ± 11 (5–38)	17 ± 11 (2–37)	0.54 (1.04)
Cadence	9 ± 7 (−5 to 20)	7 ± 5 (1–21)	9 ± 6 (0–18)	8 ± 7 (−1 to 21)	10 ± 7 (0–25)	0.55 (0.69)
Stride time	10 ± 8 (−3 to 25)	9 ± 7 (0–27)	12 ± 8 (0–22)	10 ± 10 (0–30)	12 ± 10 (−1 to 34)	0.65 (0.53)
Stride length	6 ± 11 (−9 to 33)	2 ± 6 (−8 to 12)	3 ± 9 (−12 to 24)	4 ± 6 (−3 to 15)	−1 ± 6 (−12 to 10)	0.51 (1.17)

### Physical Activity

**Figure 3:**
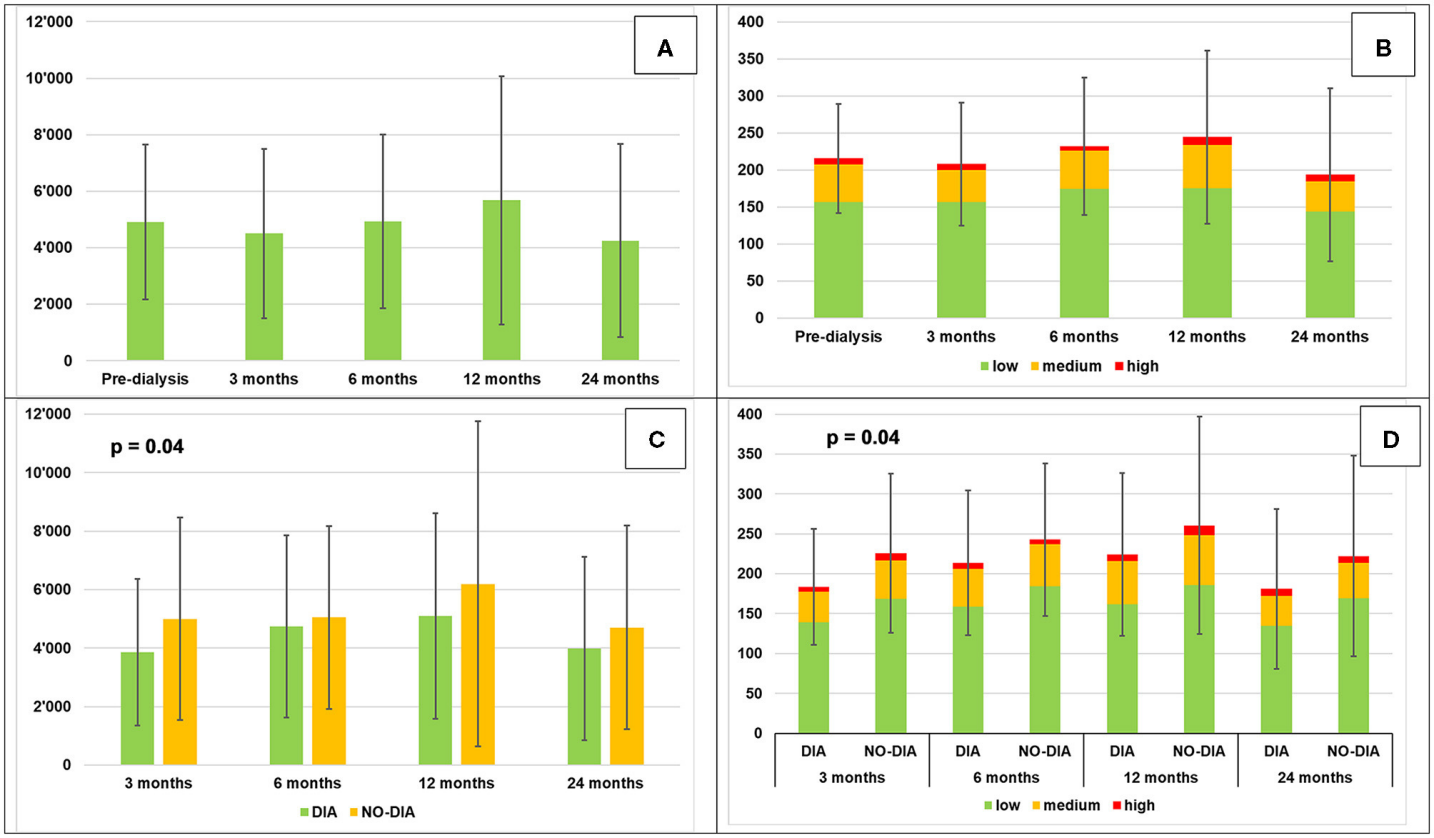
Daily physical activity (mean ± SD). **(A)** Steps per day, **(B)** Minutes of locomotion per day at different intensities, **(C)** Steps per day, dialysis vs. no-dialysis days, **(D)** minutes of locomotion per day at different intensities: dialysis vs. no-dialysis days.

At baseline, the participants walked for an average of about 3.5 h/day and did about 5000 steps/day (50% of the participants had a sedentary lifestyle with <5,000 steps/day (range 1,242–4,402) and 50% were active with >5,000 steps/day (range 5,214–11,127) (72). Once the participants started HD, they reduced their PA in the first 3 months, reached maximum PA after 1 year with >5,500 steps/day and >4 h per day, and reduced it again reaching minimum PA after 2 years with <4,500 steps/day and about 3 h/day. The percentage of the different walking intensities stayed stable throughout the study. Statistical significance was reached only for steps on the dialysis day (*p* = 0.05) and minutes at medium intensity during the dialysis day (*p* = 0.04). Comparing PA of dialysis and no-dialysis days, we found a statistical significance only at the 3-month follow-up (*p* = 0.04). Five participants at 3-month visit, 6 each for the 6- and the 12-month visit, and 4 for the 24-month visit were more active during dialysis day. Table 3 summarizes all the data that are graphically represented in Figure 3. Supplementary Material 3 reports the steps/day for each participant on dialysis and no-dialysis days.

**Table 3:** Mean physical activity in minutes and steps per day at each visit.

		**Pre-dialysis (*n* = 12)**	**3 months (*n* = 14)**	**6 months (*n* = 13)**	**12 months (*n* = 12)**	**24 months (*n* = 12)**	***P* (F)**
Steps/day	Overall	4,916 ± 2,741 (1,242–11,127)	4,500 ± 2,998 (835–10,549)	4,930 ± 3,066 (1,077–9,762)	5,676 ± 4,390 (812–15,821)	4,253 ± 3,417 (217–11,145)	0.34 (1.16)
	Dialysis day		5,004 ± 3,461 (799–11,652)^*^	5,053 ± 3,130 (1,077–10,116)	6,193 ± 5,559 (628–20,064)	4,706 ± 3,489 (420–11,751)	**0.05 (3.08)**
	No-dialysis day		3,854 ± 2,508 (1,089–9,079)^*^	4,744 ± 3,118 (1,249–11,289)	5,101 ± 3,521 (1,058–11,578)	3,990 ± 3,144 (420–10,339)	0.46 (0.77)
Min/day	Overall	216 ± 74 (80–321)	208 ± 83 (66–344)	232 ± 93 (71–385)	244 ± 117 (65–450)	194 ± 117 (24–352)	0.28 (1.36)
	Dialysis day		225 ± 100 (57—380)^**^	243 ± 96 (71–359)	261 ± 136 (52–510)	222 ± 126 (3–370)	0.23 (1.58)
	No-dialysis day		184 ± 73 (57–297)^**^	214 ± 91 (71–390)	224 ± 102 (83–390)	181 ± 100 (39–341)	0.41 (0.93)
Min at low intensity (≤ 15 steps/min)	Overall	157 ± 51 (69–234)	156 ± 51 (56–226)	174 ± 61 (55–265)	175 ± 68 (55–255)	144 ± 83 (24–276)	0.39 (1.04)
	Dialysis day		168 ± 61 (48–252)	184 ± 67 (55–257)	186 ± 74 (43–260)	169 ± 93 (3–289)	0.48 (0.82)
	No-dialysis day		139 ± 52 (48–236)	158 ± 55 (54–257)	161 ± 64 (70–253)	135 ± 72 (37–270)	0.55 (0.63)
Min at medium intensity (16–40 steps/min)	Overall	51 ± 30 (8–111)	44 ± 33 (3–97)	51 ± 35 (15–119)	59 ± 48 (10–156)	41 ± 34 (0–99)	0.21 (1.69)
	Dialysis day		48 ± 38 (3–115)	53 ± 38 (11–127)	63 ± 58 (8–179)	44 ± 39 (0–111)	**0.04 (3.99)**
	No-dialysis day		38 ± 27 (8–88)	47 ± 32 (11–108)	54 ± 39 (13–125)	38 ± 28 (1–83)	0.32 (1.17)
Min at high intensity (≥ 41 steps/min)	Overall	8 ± 12 (0–36)	8 ± 10 (0–35)	7 ± 11 (0–32)	11 ± 16 (0–56)	9 ± 14 (0–42)	0.59 (0.55)
	Dialysis day		9 ± 11 (0–35)	6 ± 10 (0–35)	13 ± 23 (0–82)	9 ± 14 (0–40)	0.66 (0.45)
	No-dialysis day		6 ± 11 (0–35)	8 ± 14 (0–45)	9 ± 15 (0–48)	9 ± 15 (0–45)	0.58 (0.48)

* *p = 0.04.*

** *p = 0.04.*

*Bold values refer the the statistical relevant results (p ≤ 0.05)*.

## Discussion

Patients on HD are known to be frailer than the general population (4, 5), but the involvement of RRT in the frailty process is not yet fully understood (10–12), although for a long time HD was thought to play an important influencing role (73, 74).

At baseline, our participants with a mean age of 72 years show a conservative gait pattern with a gait speed <1.00 m/s, a short SL (about 1.00 m), and a higher ST (about 1.20 s) that is similar to >85-year-old healthy persons. This confirms that CKD patients develop gait disorders earlier than persons with a normal renal function (41). To the best of our knowledge only two studies described spatio-temporal parameters, and DTC of gait next to gait speed in the CKD population (46, 47). One of these also reported gait movement variability in these patients (47). Shin et al. (47) reported gait movement variability values that were 33–35% greater than that of healthy age-matched individuals. Compared to these authors (CV of SL and ST <4.00 and DTC of gait speed <15%) we obtained even higher results (CV >5.00 and DTC >20%) for all measurement time points. This could be explained by the older age of our sample (mean age 72 vs. 51 years) and be due to the timing within the disease process of the baseline assessment. In fact, compared to Shin et al., who analyzed a population on HD for many years, our participants with a very low renal function had not yet received treatment, and this clinical status may have influenced gait variability parameters. In fact, at 1-year follow-up, CV (except step length) was reduced by one unit and DTC was like that in the cited studies. Gait regularity is not yet used extensively in clinical practice. Our results, however, are similar to age matched healthy persons and lower than young participants (30).

The results of the follow-up visits are interesting. Although the changes are not statistically significant, we observe a tendency toward a better locomotor functionality (better spatio-temporal parameters, decreased variability, and lower dual-task-cost), and an increase in PA (excepted only for the 3 months FU, which may be due to adaptation of the lifestyle which needs some time) in the first year of therapy, and a deterioration in the second year of HD (except DTC). This finding may suggest that HD does not negatively influence gait quality and quantity, and may even help to improve it in the first months of HD. However, further detailed investigations of gait movement performance should take place to substantiate or refute this assumption.

Regarding the quantified movement behavior, it was Interesting to see that only at the first follow-up visit the difference between dialysis and no-dialysis days was significant. In some cases, patients were more active during dialysis days, the only days they had to leave home. This is especially the case for three frail people, who leave home only if strictly necessary and which may be due to their age as well (75, 76).

DTC of gait is an indicator of cognitive functioning and CKD is known to influence cognition (77). The decrease in DTC after starting RRT that lasts for 2 years shows a possible benefit of HD in this specific cognitive aspect. Like for gait quality, further research into this aspect seems warranted and needed.

This prospective longitudinal study shows, for the first time, that there is no negative influence of HD on gait quality, and for PA in patients new to this RRT, however, the values observed indicate prevalent pathological signatures shortly before HD is initiated. The assumption seems justified that these changes emerge at earlier stages of the disease process, long before HD is initiated. The selected population that was included in a pre-dialysis program, and was, therefore, under strict medical control for years, may gain benefit from HD regarding the quality of gait, the amount of PA and attentional aspects, provided some adjustments in therapy offerings are offered at the same time. Research into the best point in time to implement effective forms of therapeutic prevention aimed at maintaining or improving gait quality is needed.

A worrying factor is that the population under investigation consistently showed high values for the variability measures of gait from the beginning of our assessments. It might be, therefore, that no deterioration was observable because of floor effects. A recent systematic review reporting optimal thresholds for movement performance (26) indicates our study participants exhibit values judged as pathological walking behavior. Such values are connected to serious negative clinical movement behavior, e.g., high incident fall rates as observed in dialysis patients (78), both before and following HD initiation (12). This finding suggests clinical gait assessment should be performed in early stages of the disease, to diagnose when gait movement performance starts to deteriorate into pathological ranges, because this would theoretically allow preventive exercise programs to be instigated.

It seems that renal disease patients have much worse gait variability in the temporal domain measures, e.g., ST, but similar characteristics in the spatial measures (SL), compared to the healthy elderly (61), to middle aged patients on HD (47), and to neurological patients (26). Different regions of the brain are associated with different spatio-temporal gait parameters (79), and in CKD mainly the pre-frontal, frontal, and temporal cortexes are affected by gray matter atrophy (80), which may explain these differences. At first sight it seems not to be logical to compare our renal disease patient data with optimal thresholds derived from healthy and neurological patients. However, although generally largely neglected in the clinic, epidemiological data suggest a higher risk of cognitive disorders and dementia in all stages of CKD (57). Impaired kidneys detrimentally affect the central nervous system, due to many CKD-specific factors that may contribute to structural and functional cerebral changes in this patient population (59). Microbleeds, augmented white matter lesions, cerebral infarcts without clinical symptoms, and silent brain infarcts, all have an increased prevalence in CKD patients (57, 81), and will negatively influence gait performance (79, 82, 83) in the form of pathological gait variability (26, 84). This could also explain the high DTC of gait we observed in our sample. Our findings are in line with a recent publication demonstrating gray matter atrophy in brain regions in control of gait and cognition in CKD patients. This study, furthermore, identified a gait phenotype specific to CKD patients that was distinct from established neurological gaits (80).

The results of this study reveal a largely impaired gait quality when discrete gait characteristics are assessed, both in pre and post HD analysis. The development of the discrete characteristics shows a similar or even better effect of HD in the sense that they do not (further) deteriorate with respect to the HD start. The discrete gait characteristics, however, are well-beyond optimal thresholds for movement performance long before HD is initiated. The assumption that CKD causes gait movement disorders by affecting different regions of the brain in earlier stages of the disease process seems reasonable. This would justify inclusion of gait analysis in an early stage of the disease process because this would allow preventive measures to mitigate the worsening of gait quality in these patients. In this regard, our findings seem to underline the importance of assessing a family of gait signatures regularly from the time CKD is diagnosed. Further studies are warranted that analyze the gait of CKD patients in clinical settings, to better understand the impact of the disease and HD on health status.

### Strength and Limitations

Although this study is one of the first that prospectively analyses gait aspects in an ESRD population in the transitional phase from pre-dialysis to a stable RRT therapy, we must draw attention to several limitations. The small sample size is due to the exploratory nature of the study performed in a geographically small Italian-speaking area of Switzerland, and the study results need to be treated as a starting point for future studies. In compliance with the aim of the study, we only recruited patients in a pre-dialysis program, therefore our findings cannot be translated to patients who were not inserted in such a program. Finally, we should mention the more advanced age of our participants compared to similar studies, which could confound the results, since age is an important factor which influences gait.

## Conclusion

Elderly patients in the pre-dialysis stage show similar spatio-temporal parameters when compared with healthy elderly and younger hemodialysis patients; however, variability was in a pathological range. This hints at changes in the central nervous system because of the kidney disease, which could also explain the higher dual-task cost of gait. Further studies with a larger sample of participants is warranted.

Patients in the pre-dialysis stage show pathological performance of gait movement variability, and this performance is not altered post-hemodialysis. This finding suggests the importance of gait analysis in early stages of CKD in the diagnosis of changes in the nervous system due to kidney disease that affect gait. Early detection of these changes would potentially allow a prevention program tailored to this population to be developed. Further studies assessing gait performance measures in all CKD stages is warranted.

## Data Availability Statement

The original contributions presented in the study are included in the article/Supplementary Material, further inquiries can be directed to the corresponding author/s.

## Ethics Statement

The studies involving human participants were reviewed and approved by the regional ethics committee Comitato etico del Canton Ticino that approved the trial with ID number 2019-01161, CE 3497. The patients/participants provided their written informed consent to participate in this study.

## Author Contributions

DZ designed the study, collected and analyzed the data, and wrote the manuscript. OG designed the study, recruited patients, collected data, and contributed to the writing of the manuscript. EB helped to design the study and contributed to the data analysis and the writing of the manuscript. PQ and MT designed the study and contributed to the writing of the manuscript. MR contributed to the data analysis and to the writing of the manuscript. All authors contributed to the article and approved the submitted version.

## Conflict of Interest

DZ received financial support from ABREOC for his doctoral thesis this article belongs to. The remaining authors declare that the research was conducted in the absence of any commercial or financial relationships that could be construed as a potential conflict of interest.

## Publisher's Note

All claims expressed in this article are solely those of the authors and do not necessarily represent those of their affiliated organizations, or those of the publisher, the editors and the reviewers. Any product that may be evaluated in this article, or claim that may be made by its manufacturer, is not guaranteed or endorsed by the publisher.

## Footnotes

^1^Estimated Glomerular Filtration Rate.
